# Parental divorce in childhood does not independently predict maternal depressive symptoms during pregnancy

**DOI:** 10.1186/s12884-020-03227-w

**Published:** 2020-09-07

**Authors:** Elviira Porthan, Matti Lindberg, Eeva Ekholm, Noora M. Scheinin, Linnea Karlsson, Hasse Karlsson, Juho Härkönen

**Affiliations:** 1 FinnBrain Birth Cohort Study, Turku Brain and Mind Center and Centre for Population Health Research, Department of Clinical Medicine, University of Turku, Lemminkäisenkatu 3, 20520 Turku, Finland; 2 Faculty of Social Sciences, Department of Social Research, University of Turku, Turku, Finland; 3 Department of Obstetrics and Gynecology, Turku University Hospital and University of Turku, Turku, Finland; 4 Department of Psychiatry, Turku University Hospital and University of Turku, Turku, Finland; 5 Department of Child Psychiatry, Turku University Hospital and University of Turku, Turku, Finland; 6 Centre for Population Health Research, University of Turku and Turku University Hospital, Turku, Finland; 7 Department of Political and Social Sciences, European University Institute, Firenze, Italy; 8 Department of Sociology, Stockholm University, Stockholm, Finland

**Keywords:** prenatal depression, depression, parental divorce, family conflicts, domestic violence

## Abstract

**Background:**

This study sought to investigate if parental divorce in childhood increases the risk for depressive symptoms in pregnancy.

**Methods:**

Women were recruited during their ultrasound screening in gestational week (gwk) 12. The final study sample consisted of 2,899 pregnant women. Questionnaires (including the Edinburgh Postnatal Depression Scale) were completed at three measurement points (gwk 14, 24 and 34). Prenatal depressive symptoms were defined as Edinburgh Postnatal Depression Scale score ≥ 13. Parental divorce and other stressful life events in childhood were assessed at gwk 14. Parental divorce was defined as separation of parents who were married or cohabiting. Questionnaire data was supplemented with data from Statistics Finland and the Finnish Medical Birth Register.

**Results:**

Parental divorce in childhood increased the risk for depressive symptoms during pregnancy (OR 1.47; 95% CI 1.02–2.13), but the connection was no longer significant after adjusting for socioeconomic status, family conflicts and witnessing domestic violence in the childhood family (OR 0.80; 95% CI 0.54–1.18).

**Conclusions:**

Parental divorce alone does not predict depressive symptoms during pregnancy.

## Background

Depression affects 7–20% of women at some point during pregnancy [1–3]. It is associated with an increased risk of postpartum depression [4], increased number of birth complications, such as premature labour [5] and low offspring birth weight [6] as well as negative health behaviours during pregnancy, such as smoking and substance abuse [7–10]. Furthermore, children whose mothers were depressed during pregnancy are themselves at an increased risk of depression later in life [11]. Besides impairment of subjective well-being, depression is a major economic burden: lifetime costs of perinatal depression have been estimated to be around £76,000 per woman in the United Kingdom [12].

Prenatal depression often goes undiagnosed [13]. Identifying predisposing factors can help to identify women at risk of depression. Previous research has found that predisposing factors include history of depression [14, 15], traumatic and other adverse childhood events [16, 17], life stress, unintended pregnancy, lack of social support, domestic violence, low income [15] and single marital status [18, 19].

This is among the first studies to investigate whether parental divorce predicts prenatal depressive symptoms. Parental divorce and in-family conflicts are common childhood experiences, and both are predictors of depression in adulthood [20–22]. However, for three reasons it is unclear whether the association is found among pregnant women. First, pregnancy is a transitional life course stage often associated with positive emotions [23]. Second, pregnant women might experience more social support than non-pregnant women [24], which could protect them from depression [1]. Third, depressed women seem to have fewer children overall [25]. Thus, we asked whether the association between parental divorce and depression in the general population is present among pregnant mothers as well. If parental divorce does predict prenatal depression, this association may have long-term consequences to childhood conditions that span to the next generation.

## Methods

### Study population and data collection

This study included women from the FinnBrain Birth Cohort Study (www.finnbrain.fi). The aim of the FinnBrain study is to investigate the effect of environmental and genetic factors on later child health outcomes. 3,808 women living in Southwest Finland or the Åland islands were enlisted between December 2011 and April 2015 during their ultrasound screening in gestational week (gwk) 12. Women who participated in first trimester screening ultrasound and who had sufficient Finnish or Swedish language skills and normal screening results were approached by research nurses. The ultrasound is offered to all pregnant women by public healthcare and the participation rate is high. The FinnBrain questionnaire data was linked to data from Statistics Finland and the Finnish Medical Birth Register (FMBR), maintained by the Finnish National Institute for Health and Welfare (NIHW) (www.thl.fi).

Our sample consisted of 3,048 cases (80% of mothers enlisted to the FinnBrain study), who answered the first questionnaire in gwk 14. We excluded mothers born outside Finland (n = 97). In addition, women with missing information on country of birth (n = 28) were excluded due to incomplete or missing register data. We further excluded women without the Edinburgh Postnatal Depression Scale (EPDS) score at any of measurement (see below) timepoints (n = 24). The final sample size was 2,899 mothers.

### Measures

Participants completed self-reported questionnaires in gwks 14, 24 and 34 as well as three months postpartum [26]. Prenatal depressive symptoms were assessed with the ten-item EPDS questionnaire, which has been validated to screen prenatal depression and has proven highly reliable [27]. Each question is scored from 0 to 3 points and the total score ranges from 0 to 30 points. We define prenatal depressive symptoms as depressive symptoms during pregnancy (EPDS score ≥ 13 at least once during pregnancy), even if the symptoms started before the pregnancy. We used 13 points as a cut-off value for depressive symptoms, as that value is used in Finnish maternity care to evaluate depressive symptoms by a physician [28]. EPDS was collected at each of the three measurement points during pregnancy as well as three months postpartum.

Parental divorce, family conflicts and domestic violence in childhood were assessed with questionnaires at gwk 14. The questions assessed life events in three age categories (0–6 years, 7–12 years and 13–18 years). The question on parental divorce during childhood was binary (yes/no). Divorced parents included parents who were married or cohabiting before the separation. Questions on parental conflicts and domestic violence were answered on a Likert scale from 0 to 4 (0 = never, 1 = rarely, 2 = sometimes, 3 = often, and 4 = almost always). We used the highest score reported in any of the age categories. The question on conflicts in the childhood family was modified from the Health 2000 questionnaire [29, 30].

Information on the socioeconomic status of the childhood family was obtained from Statistics Finland registers. It referred to the occupation of the childhood household’s reference person, and was divided into four categories (1. upper-level employees, 2. lower-level employees and self-employed, 3. manual workers, and 4. students, unemployed, pensioners and others). Information on parity came from the FMBR. This register data was linked to the FinnBrain cohort data.

### Statistical analyses

Differences in the EPDS scores between women whose parents had or had not divorced before age 18 were analysed with a two-sample two-tailed t-test. The association between parental divorce and prenatal depressive symptoms was analysed with multilevel random effects logistic regression. The effect of age category (0–6 years, 7–12 years and 13–18 years) in which the parental divorce took place on EPDS scores was analysed with a Kruskal-Wallis H-test (additional analysis, data not shown). All mothers with at least one EPDS score from any measurement point were included in the multilevel logistic regression analysis. Multilevel logistic regression can handle such nested and unbalanced data. Additionally, it takes into account the correlation between responses by the same mother. Multilevel modeling was used in order to increase the sample size and to reduce measurement error due to variability in responses between the measurement points.

We estimated two multilevel logistic regression models, where depressive symptoms—defined as an EPDS score of 13 or above—was the outcome variable and the main independent variable was the divorce of the parents of pregnant women before child age 18. In Model 1, the control variables were parity and age during pregnancy. Model 2 added childhood socioeconomic status (the socio-economic group of the childhood household reference person) and negative childhood experiences (dummy variables conflicts and witnessing domestic violence) as additional confounders, as the effect of the divorce is likely to be confounded by family conditions.

Missing values in explanatory variables (due to non-response in the first questionnaire), including questions on parental divorce and negative life events in childhood, were imputed using STATA’s simulation based multivariate imputation by chained equations (MICE) method [31]. MICE allows for imputation models to be tailored to the level of measurement for each variable: the present study used ordinal and logistic regression models. In total, twenty simulated imputations were created for each missing response. The EPDS score consisted of a sum of ten question responses, collected at each measurement point. When no more than three of these answers were missing due to non-response, these missing items were imputed within each measurement point by using the average of the non-missing items. When more than three answers in each measurement point were missing, the complete EPDS score was classified as missing. Missing dependent variables can be handled by the multilevel random effects models used in the analyses [31]. Therefore, EPDS scores did not require further imputation.

The final sample size was 2,899 mothers. Each woman had at least one EPDS score. The number of complete cases was 2,074.

Additionally, interaction analysis was used to investigate the interaction between parental divorce and witnessing domestic violence or serious conflicts in the family. A likelihood-ratio test (with non-imputed data) with and without the interaction was used to compare the model fit (additional analyses, data not shown).

### Statistical analyses were carried out using STATA version 14.2

This study was funded by the Academy of Finland (decision numbers 134,950, 253,270, 287,908 and 324,613), the Valto Takala Fund, Signe and Ane Gyllenberg Foundation and Finnish State Grants for Clinical Research (ERVA). The funders were not involved in the analysis or writing of the manuscript.

## Results

The overall response rates were 81% in gwk 14, 73% in gwk 24 and 69% in gwk 34. The mean age of the women was 31 years and 52.4% of them were nulliparous. The parents of 27.8% of the expecting women had divorced before her age of 18. Sociodemographic factors and negative events during the pregnant women’s childhood are presented in Table 1.

**Table 1:** Sociodemographic factors and negative events during childhood of pregnant women who had EPDS scores below the cut-off score of 13 or equal to / over the cut-off

	EPDS < 13	EPDS ≥ 13	*P*-value of χ2 test
	n (%)	n (%)	
Parity			0.017
Nullipara	1 377 (53.1%)	141 (45.9%)	
Multipara	1 215 (46.9%)	166 (54.1%)	
Age			0.110
≤ 20 years	37 (1.5%)	8 (2.7%)	
21–25 years	311 (12.3%)	44 (14.6%)	
26–30	946 (37.4%)	95 (31.6%)	
31–35	909 (36.0%)	109 (36.2%)	
36–40	294 (11.6%)	38 (12.6%)	
≥ 41 years	30 (1.2%)	7 (2.3%)	
Socioeconomic status when the mother was aged 15 years or under			0.300
Upper-level employee	709 (28.9%)	73 (25.8%)	
Lower-level employee or self-employed	1 051 (42.9%)	138 (48.8%)	
Manual worker	570 (23.2%)	60 (21.2%)	
Student, unemployed, pensioner or other	123 (5.0%)	12 (4.2%)	
Parents divorced before age 18			0.064
Yes	695 (27.3%)	97 (32.3%)	
No	1854 (72.7%)	203 (67.7%)	
Witnessed domestic violence in childhood family (in example, between parents): highest value at age 0–18 years			< 0.001
Never	1 998 (78.1%)	185 (61.7%)	
Very rarely	244 (9.5%)	38 (12.7%)	
Sometimes	196 (7.7%)	48 (16.0%)	
Often	84 (3.3%)	17 (5.7%)	
Very often	37 (1.5%)	12 (4.0%)	
Serious conflicts in childhood family: highest value at age 0–18 years			< 0.001
Never	1 322 (51.7%)	94 (31.5%)	
Very rarely	465 (18.2%)	58 (19.5%)	
Sometimes	439 (17.2%)	72 (24.2%)	
Often	249 (9.7%)	48 (16.1%)	
Very often	83 (3.2%)	26 (8.7%)	

Non-imputed data were used for all calculations

**Fig. 1:**
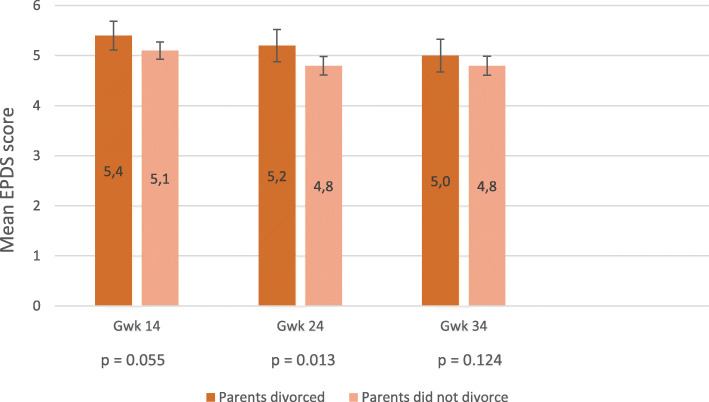
The effect of parental divorce during the women’s childhood on mean EPDS scores in pregnancy (gwk = gestational weeks)

**Fig. 2:**
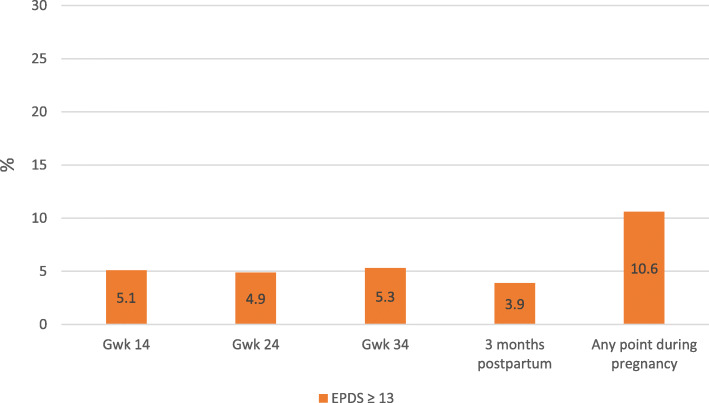
The prevalence of EPDS score ≥ 13 in pregnancy (gwk=gestational weeks)

The mean EPDS scores are shown in Fig. 1. The prevalence of depressive symptoms defined as EPDS score ≥ 13 are presented in Fig. 2. The association between parental divorce and the mean EPDS scores and respective p-values of two-tailed t-tests at different measurement points are presented in Fig. 1. Additional analyses (not shown) using the Kruskall-Wallis H-test found no evidence that the association between parental divorce and EPDS scores would be stronger at some childhood stages than others (0–6 years, 7–12 years, or 13–18 years).

Multilevel logistic regression analysis (Table 2) showed that women whose parents had divorced were more likely to have depressive symptoms at any point during pregnancy than women from intact families (OR 1.47; 95% CI 1.02–2.13) when controlled for maternal age and parity (Model 1). After controlling for childhood socioeconomic status and the selected adverse events in childhood (Model 2), the association between parental divorce and depressive symptoms attenuated and were not statistically significant (OR 0.80; 95% CI 0.54–1.18). Serious family conflicts and witnessing domestic violence in the childhood family increased the risk for prenatal depressive symptoms (OR 1.54; 95% CI 1.31–1.80 for conflicts; OR 1.27; 95% CI 1.06–1.53 for witnessing domestic violence).

**Table 2:** Parental divorce and prenatal depressive symptoms: multilevel logistic regression

	Model 1	Model 2	
	**aOR (95% CI)**		**aOR (95% CI)**
**Parental divorce**	**1.47 (1.02–2.13)**		0.80 (0.54–1.18)
**Measurement points during pregnancy**			
Gwk 14	ref.		ref.
Gwk 24	0.96 (0.72–1.29)		0.96 (0.72–1.28)
Gwk 34	1.09 (0.81–1.45)		1.08 (0.81–1.45)
**Maternal age**	0.98 (0.94–1.02)		0.96 (0.93–1.00)
**Parity**			
Multiparous	ref.		ref.
Nulliparous	**0.60 (0.42–0.85)**		**0.62 (0.44–0.87)**
**Socioeconomic status in childhood**			
Upper level employee			ref.
Lower level employees and self-employed			1.40 (0.92–2.14)
Manual workers			1.08 (0.66–1.78)
Students, unemployed, pensioners and others			1.37 (0.59–3.21)
**Serious conflicts in the childhood family**			**1.54 (1.31–1.80)**
**Witnessed domestic violence in the childhood family**			**1.27 (1.06–1.53)**

Missing variables of childhood socioeconomic status, serious conflicts, witnessing domestic violence and parental mental health problems were imputed in multilevel logistic regression analysis. Number of missing values: EPDS score in first measurement point 29; in second measurement point 410; in third measurement point 543; parental divorce 50; maternal age 0; parity 0; childhood socioeconomic station 163; serious conflicts in childhood family 43; witnessing domestic violence in childhood family 40

Likelihood-ratio tests indicated that adding the interaction between conflicts and parental divorce (*p* = 0.679) or witnessing domestic violence and parental divorce (*p* = 0.651) did not improve the model fit over model 2 (non-imputed data was used when calculating likelihood-ratio tests).

Socioeconomic status of the childhood family or maternal age during pregnancy were not associated with prenatal depressive symptoms. The risk for depressive symptoms was lower in nulliparous women in both models.

The imputation of missing values in explanatory variables (in multilevel logistic regression analysis) had a negligible impact on the results, and the main findings remained unchanged.

## Discussion

Parental divorce was associated with an elevated risk of prenatal depressive symptoms when controlled for maternal age and parity. After adjusting for childhood living conditions (childhood socioeconomic status, conflicts and witnessing domestic violence), the association was no longer significant.

Parental divorce has been found to predict depression in adulthood [21], and in the general population divorce remains a risk for depression after adjusting for childhood socioeconomic status [32, 33]. Our results show that, in contrast to many studies in general adult populations, there is no association between parental divorce and depressive symptoms during pregnancy after controlling for socioeconomic class, conflicts, and violence in the childhood family.

There are several possible reasons for the different effect of parental divorce in pregnant women compared to the general population. First, pregnant women may have a smaller risk of depression, as pregnancy is generally considered a positive life event [23]. Second, pregnant women might experience more social support than non-pregnant women [24], which could also protect them from depression [1]. Third, depressed women may be less likely to become pregnant [25]. These reasons could weaken an association between parental divorce and prenatal depression. Fourth, measures of conflict and violence in the childhood family were controlled for in the analyses of this study in contrast to some previous studies [21]. Conflicts and violence are associated with impaired psychological well-being both in childhood [34, 35] and later in adulthood [36], and may confound the relationship between parental divorce and prenatal depression. Further, conflicts and violence are often reported to account for a reduced psychological well-being in children before parental divorce occurs [37, 38]. Conflict and violence in the childhood family may also mediate the relationship between parental divorce and prenatal depressive symptoms if they occur as a part of the parental divorce process that stretches beyond the physical divorce of the parents.

Nulliparous women had a lower risk for depressive symptoms in the present study, as previously reported by our research group [39]. Interestingly, childhood socioeconomic status was not associated with depressive symptoms, whereas conflicts and witnessing domestic violence in the childhood family were. In line with our results during pregnancy, previous studies have reported interparental conflicts and witnessing domestic violence in childhood to be associated with depression in adulthood [36, 40].

Strengths and limitations.

One of the strengths of the present study was that we received data from multiple registers. In addition, data on depressive symptoms were assessed repeatedly during pregnancy. Response rates were high, but decreased by the third trimester (69%). Mothers who responded to the questionnaires both in gwks 14 and 34 had fewer depressive symptoms, were older, more often nulliparous and were more highly educated than those who did not respond to the questionnaire in gwk 34 [26]. However, our estimate of the prevalence of depressive symptoms (point prevalence of 4.9–5.3% and period prevalence of 10.6%) was comparable to previous findings. The prevalence has been estimated to be 7–20% [1–3] and the point prevalence 7.7% in Finland [41].

Mothers suffering from more severe depression may have been less likely to participate in the study. Women more susceptible to or already suffering from depression are more likely to discontinue participating in the study, as multiple follow-up questionnaires during pregnancy and after birth may be more laborious for depressed mothers.

Negative events during childhood were inquired (retrospectively) throughout childhood, not only regarding the time of the divorce. Therefore, the answers might represent negative experiences after the divorce as well. In addition, recall bias is a known limitation: even though recall bias is less prominent in retrospective assessments of domains of strong emotional content [42], it is unlikely to be completely absent, and current depression could further distort childhood experiences towards the negative.

Besides the covariates used in our analyses, there are other, well-known risk factors for prenatal depression. Many of these, such as social support or a history of depression [26], were not measured in our data. However, these measures as well as factors such as maternal education are temporarily preceded by parental divorce and therefore likely mediators of an effect of parental divorce. Including them could have biased the results by diminishing a true effect. Whether pregnancy weakens the association between parental divorce and depression or whether depressed mothers are less likely to become pregnant is an interesting question for future researchers with access to appropriate longitudinal data. Although we had information on marital status, it was not included it in the covariates due to the small proportion of single mothers (1.7%). We did not have reliable information on depression before the pregnancy. However, our aim was to focus on the presentation of depressive symptoms during pregnancy as such, even if they had started before the pregnancy.

Although parental divorce during childhood and depression later in life are associated, the magnitude of the effect varies between studies, the causality is controversial and it is likely that several confounding factors remain. Further, while parental divorce may be a negative experience to some, it can be a relief to others.

## Conclusions

This is among the first studies to explore the relationship between parental divorce and prenatal depressive symptoms. Although parental divorce increases the risk of depression in non-pregnant populations, it did not predict prenatal depression. In contrast, serious family conflicts and witnessing domestic violence in the childhood family increased the risk for prenatal depressive symptoms in this low risk cohort. Information of negative childhood living conditions could help better recognize depressed women during pregnancy.

## Data Availability

Due to Finnish federal legislation, the research data cannot be made available online, but data can potentially be shared via Material Transfer Agreement as part of research collaboration. Requests for collaboration can be sent to the Board of the FinnBrain Birth Cohort Study; please contact PI Prof Hasse Karlsson (hasse.karlsson@utu.fi) and co-PI Adj Prof Linnea Karlsson (linnea.karlsson@utu.fi).

## Abbreviations

Gwk: Gestational week
EPDS: Edinburgh Postnatal Depression Scale
NIHW: Finnish National Institute for Health and Welfare
FMBR: Finnish Medical Birth Register
MICE: Multivariate imputation by chained equations
